# Dental impact of anti-fibroblast growth factor 23 therapy in X-linked hypophosphatemia

**DOI:** 10.1038/s41368-023-00259-8

**Published:** 2023-12-06

**Authors:** Elis J. Lira dos Santos, Kenta Nakajima, Julien Po, Ayako Hanai, Volha Zhukouskaya, Martin Biosse Duplan, Agnès Linglart, Takashi Shimada, Catherine Chaussain, Claire Bardet

**Affiliations:** 1 Université Paris Cité, Institut des maladies musculo-squelettiques, Laboratory Orofacial Pathologies, Imaging and Biotherapies URP2496 and FHU-DDS-Net, Dental School, and Plateforme d’Imagerie du Vivant (PIV), Montrouge, France; 2 R&D Division, Kyowa Kirin, Co., Ltd, 3-6-6 Asahi-machi, Machida-shi, Tokyo, Japan; 3 Université Paris Cité, Institut Imagine, INSERM UMR 1163, Paris, France; 4 AP-HP, Reference Center for Rare Disorders of the Calcium and Phosphate Metabolism, Dental Medicine Department, Bretonneau Hospital, GHN-Université Paris Cité, Paris, France; 5 Paris-Saclay University, AP-HP, INSERM U1185, DMU SEA, Endocrinology and Diabetes for Children, Reference Center for Rare Diseases of the Calcium and Phosphate Metabolism, OSCAR filière, EndoRare, and BOND ERNs, Bicêtre Paris Saclay Hospital, Le Kremlin-Bicêtre, France; 6 Medical Affairs Department, Kyowa Kirin, Co., Ltd, 1-9-2 Otemachi, Chiyoda-ku, Tokyo, Japan

**Keywords:** Periodontitis, Calcium and vitamin D, Translational research

## Abstract

Elevated fibroblast growth factor 23 (FGF23) in X-linked hypophosphatemia (XLH) results in rickets and phosphate wasting, manifesting by severe bone and dental abnormalities. Burosumab, a FGF23-neutralizing antibody, an alternative to conventional treatment (phosphorus and active vitamin D analogs), showed significant improvement in the long bone phenotype. Here, we examined whether FGF23 antibody (FGF23-mAb) also improved the dentoalveolar features associated with XLH. Four-week-old male *Hyp* mice were injected weekly with 4 or 16 mg·kg^−1^ of FGF23-mAb for 2 months and compared to wild-type (WT) and vehicle (PBS) treated *Hyp* mice (*n* = 3–7 mice). Micro-CT analyses showed that both doses of FGF23-mAb restored dentin/cementum volume and corrected the enlarged pulp volume in *Hyp* mice, the higher concentration resulting in a rescue similar to WT levels. FGF23-mAb treatment also improved alveolar bone volume fraction and mineral density compared to vehicle-treated ones. Histology revealed improved mineralization of the dentoalveolar tissues, with a decreased amount of osteoid, predentin and cementoid. Better periodontal ligament attachment was also observed, evidenced by restoration of the acellular cementum. These preclinical data were consistent with the retrospective analysis of two patients with XLH showing that burosumab treatment improved oral features. Taken together, our data show that the dentoalveolar tissues are greatly improved by FGF23-mAb treatment, heralding its benefit in clinics for dental abnormalities.

## Introduction

X-linked hypophosphatemia (XLH) is the most common cause of genetic rickets (1/20 000).^1^ XLH occurs due to inactivating mutations of Phosphate-regulating Endopeptidase Homolog X-linked (PHEX),^2^ resulting in increased circulating levels of Fibroblast Growth Factor 23 (FGF23), a bone-derived hormone that leads to renal phosphate wasting and inhibition of the endogenous synthesis of 1,25-(OH)_2_-vitamin D_3_. XLH manifests by short stature, leg bowing, bone pain, craniosynostosis, osteomalacia, higher prevalence of overweight and early obesity, and spontaneous dental abscesses.^1,3,4^ With aging, patients develop musculoskeletal pain and fatigue, osteoarthritis, pseudofractures, enthesophytes, hyperparathyroidism, progressive deafness and several oral manifestations including endodontic infections and higher susceptibility to periodontitis.^5,6^

Oral manifestations in children and adults with XLH mainly result from abnormal dentin, cementum and alveolar bone mineralization as previously reported in humans^4,6–8^ and in the *Hyp* mouse model, the most studied model of the disorder.^9,10^ The conventional treatment, which aims to counteract the consequences of FGF23 excess, consists of oral phosphorus supplementation with multiple daily intakes to compensate for renal phosphate wasting and active vitamin D analogs, to counter the 1,25-(OH)_2_-vitamin D_3_ deficiency.^11^ This treatment is commonly prescribed from early childhood to the end of growth.^5,12^ The outcomes of the conventional therapy vary depending on disease severity, age at start of treatment, treatment regimen and treatment adherence.^5,11^ In addition, it has been shown to improve oral manifestations by correcting dentin and cementum mineralization.^6,7^

A recent and promising strategy conducted in clinical trials in children and adult patients downregulates FGF23 signaling using a monoclonal antibody against FGF23, called burosumab. In *Hyp* mice, anti-FGF23 therapy increases Cyp27b1 expression and suppresses Cyp24a1.^13^ Consequently, in treated *Hyp* mice, serum phosphate levels and 1,25-(OH)_2_-vitamin D_3_ are restored.^13,14^ Phase I, II and III studies provided the safety and effectiveness incentives of this approach^15–18^ leading thus to the approval of this treatment by the Food and Drug Administration in USA, by the European Medical Agency in Europe and by some national agencies. In some countries, burosumab is now prescribed for certain conditions for children and adults affected by XLH.^12^ The therapy using FGF23 antibody was shown to improve growth and long bone osteomalacia in *Hyp* mice,^13^ and in humans.^17,19,20^ Our group recently reported a decrease in the incidence of dental abscesses in XLH children treated with burosumab for an average of 3.2 years compared to conventional treatment.^21^ However, a prospective case control study involving 10 children with XLH showed the persistence of enlarged pulp chambers, a hallmark feature of XLH, after 3 years of burosumab.^22^

Furthermore, a preclinical study showed that treatment with a FGF23 antibody (Amgen) in *Hyp* mice had limited benefits on oral features.^23^ Therefore, the impact of burosumab on dento-alveolar tissues is still matter of debate whereas this therapy is nowadays frequently prescribed.^12,24,25^ Here, we aimed to determine whether FGF23 antibody (FGF23-mAb) therapy improved the dental features associated with XLH in young *Hyp* mice.

## Results

### Burosumab treatment in patients with XLH improves alveolar bone and dental features

**Fig. 1:**
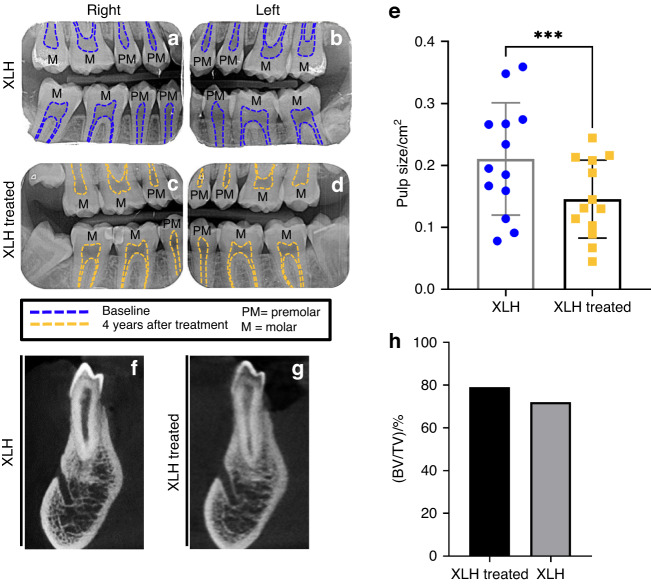
Improved pulp chamber size and bone features in patients with XLH treated with burosumab. **a**–**d** Representative periapical radiographs of a 20-y-old female with XLH treated with burosumab for 4 years. Blue dotted lines delimitate pulp chamber; Yellow dotted lines highlight reduced pulp chamber sizes after treatment. **e** Measurements of pulp chamber. **f**, **g** Representative images of CBCT in the mandibular premolar region from a 49-year-old male with XLH before and after treatment with burosumab for 6 years. **h** Bone fraction (BV/TV) at baseline and following treatment

We analyzed clinical data of two patients with XLH treated with burosumab for 4 and 6 years. The first patient, a 20-year-old female, was treated since the diagnosis at the age of 1 year with calcitriol and phosphorus, which was replaced by burosumab at the age of 16. At the onset of burosumab treatment, the patient exhibited enlarged pulp chambers. Measurements of pulp chamber size following this treatment showed a positive outcome on these features (Fig. 1a–e). The second patient, a 49-year-old male, was treated with calcitriol and phosphorus from 3 to 18 years of age. He resumed conventional treatment at 37 years of age, which was replaced by burosumab at 43 years of age. Evaluation of the bone fraction (BV/TV) showed an improvement during the time of burosumab treatment (Fig. 1f–h).

### Dentinomalacia and enlarged pulp chambers are corrected by FGF23-mAb treatment in *Hyp* mice

**Fig. 2:**
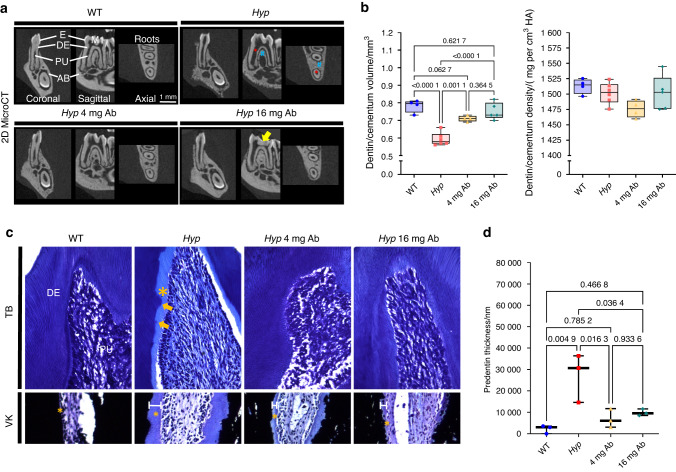
Impact of FGF23-mAb therapy on dentin in 3-month-old *Hyp* mice. **a** Two-dimensional micro-computed tomography (mCT) shows mandibular first molar enamel (E), dentin (DE), pulp chamber (PC) and alveolar bone (AB). *Hyp* mice present enlarged pulp chamber (red asterisk), thin dentin and altered alveolar bone (blue hashtag). Treatment shows dentin formation (yellow arrow) with 4 or 16 mg·kg^−1^ of FGF23-mAb. **b** Both concentrations of FGF23-mAb restored dentin/cementum volume, the higher concentration resulting in a rescue similar to WT levels. **c**, **d** Toluidine blue (TD) staining (crown region, upper panel) reveals wide predentin (PD) (yellow *) and erratic PD-DE border with interglobular DE patterns in vehicle treated *Hyp* mice (yellow arrows). DE and PD are fully normalized by FGF23-mAb treatment at a dosage of 4 mg·kg^−1^ and 16 mg·kg^−1^. Von Kossa (VK) staining (root region) highlights the reduced predentin and improved calcospherite fusion during dentin mineralization in 3 months old FGF23 treated *Hyp* mice

We next explored the impact of the FGF23-mAb treatment in *Hyp* mice to assess whether the positive outcomes found on alveolar bone and dental features in two adult patients with XLH treated with burosumab was also found in the mouse model of the disorder (Fig. 1). Micro-CT analyses showed that both doses of FGF23-mAb treatment significantly improved the dentin/cementum volume when compared to vehicle-treated animals (*P* = 0.001 1 for 4 mg·kg^−1^ and *P* < 0.000 1 for 16 mg·kg^−1^), reaching a volume comparable to WT (Fig. 2a, b). In contrast, no difference in terms of dentin/cementum density was found between the different groups.

**Fig. 3:**
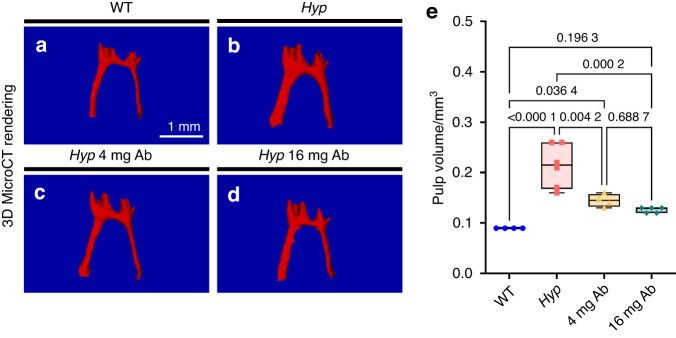
Impact of FGF23-mAb therapy on pulp in *Hyp* mice. **a**–**e** Three-dimensional micro-computed tomography (mCT) shows that enlargement of pulp chambers was also improved with a reduced pulp volume in treated *Hyp* mice

We confirm micro-CT findings through histology on the 3-months old mice, showing that *Hyp* mice displayed abnormal first molar including thin dentin and widened predentin (Fig. 2c). Toluidine blue and Von Kossa stainings revealed enlarged predentin (PD) and erratic predentin-dentin border with interglobular dentin patterns in the molar crown and root in the same *Hyp* mice (Fig. 2c). In contrast, dentin mineralization and predentin width were fully restored in animals treated with the FGF23-mAb, whatever the dose (Fig. 2d). Enamel parameters measured in molars were not significantly different between genotypes or treatment groups (Supplementary Fig. 1). Same parameters through micro-CT analysis in the continuously growing mandibular incisors did not show significant differences between the 3 groups (Supplementary Fig. 2). Enlargement of pulp chambers was also restored in FGF23-mAb treated Hyp mice when compared with vehicle-treated *Hyp* mice (*P* = 0.004 2, *P* = 0.000 2, respectively) (Fig. 3a–e), which is consistent with the amelioration of pulp chamber size in the XLH patient treated with burosumab. Quite remarkably, the 16 mg·kg^−1^ dose allowed a complete recovery of the volume of the pulp chamber, reaching values similar to that of the WT mice (0.126 versus 0.09 mm^3^, *P* = 0.196 3). Taken together, our data show that repeated treatment with FGF23-mAb at 4 mg·kg^−1^ or 16 mg·kg^−1^ improves the dentin mineralization and the pulp volume in young *Hyp* mice; interestingly, the higher concentration results in a dentoalveolar rescue similar to the WT animals.

### FGF23-mAb treatment improves the tooth anchorage apparatus in *Hyp* mice

**Fig. 4:**
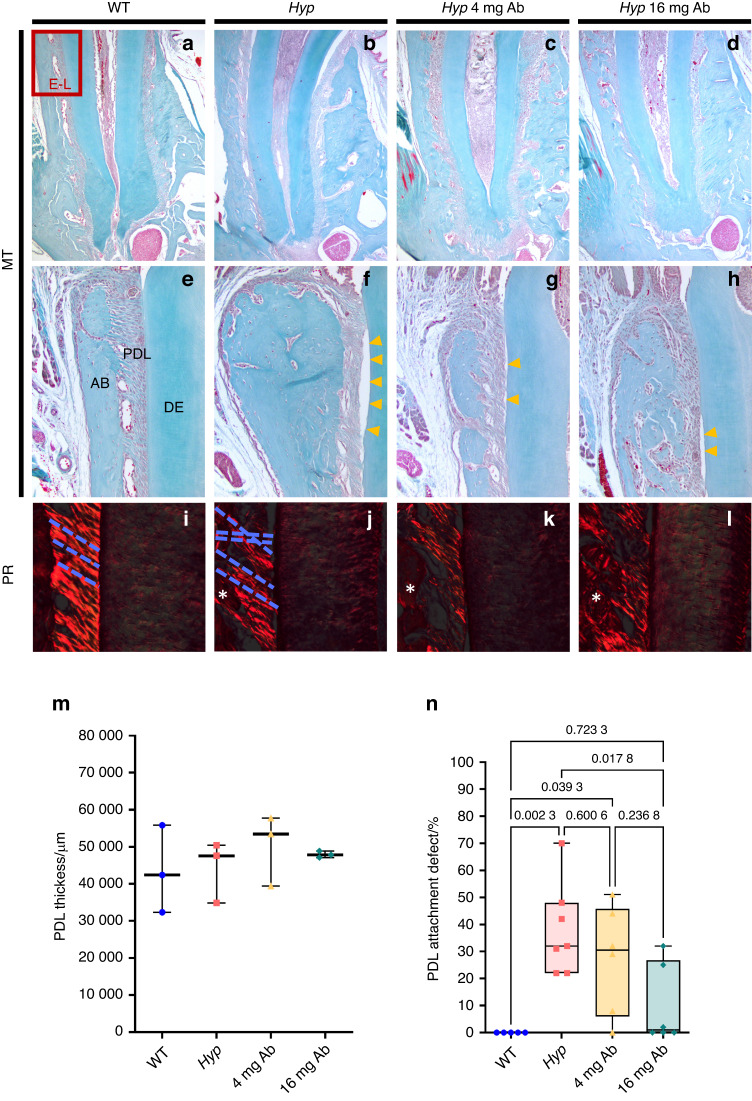
Histological analyses of the impact of FGF23-mAb therapy on the periodontal ligament in 3-month-old *Hyp* mice. **a**–**h** Masson’s Trichrome (MT) staining revealed decreased periodontal ligament attachment in *Hyp* mice. **f**–**h** Yellow arrowheads indicates defective Periodontal ligament (PDL) attachment. **i**–**l** Picrosirius red (PR) staining viewed under polarized light microscopy emphasizes highly organized periodontal ligament (PDL) fibers in wild-type (WT) mice (blue dotted line). **m**, **n** *Hyp* mice have a loss of PDL fiber organization and no alteration in PDL thickness, and higher dose treatment improved functional attachment between acellular cementum (AC) and alveolar bone (AB)

**Fig. 5:**
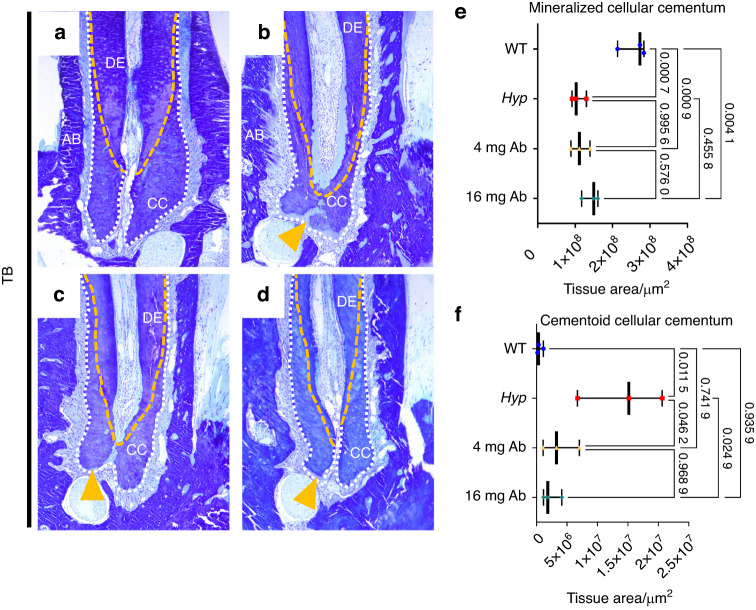
Histological analyses of the impact of FGF23-mAb therapy on cellular cementum in 3-month-old *Hyp* mice. **a**–**d** Cementoid accumulation is observed in *Hyp* mice compared to WT mice (yellow arrow). **e**–**f** Histomorphometry confirms significant effect of FGF23-mAb with complete rescue of cellular cementum mineralization

Periodontitis is frequently reported in adult patients with XLH, even in young adults, mainly resulting from impaired tooth anchorage apparatus.^6,9^ Impact of FGF23-mAb on periodontium in *Hyp* mice was investigated as well by micro-CT and histology (Fig. 4). Masson’s trichrome highlighted altered attachment of the periodontal ligament (PDL) in 3-month-old vehicle-treated *Hyp* mice (Fig. 4e–h); this specific feature was only partially present in FGF23-mAb treated mice. Picrosirius red staining under polarized light revealed highly disorganized PDL in *Hyp* mice (Fig. 4j) when compared to WT animals. Interestingly, in both groups of FGF23-mAb treated *Hyp* mice, this attachment was improved, as demonstrated by the functional orientation of the fibers together with no alteration in PDL thickness (Fig. 4i–l, m); this effect was more marked in the group of animals that received the 16 mg·kg^−1^ dose of FGF23-mAb (Fig. 4n). *Hyp* mice showed an increased amount of non-mineralized matrix in the cellular cementum in comparison with WT and FGF23-mAb treated mice (Fig. 5a–d). Quantification further revealed a complete rescue of the cellular cementum mineralization in mice treated with both FGF23-mAb doses (Fig. 5e, f).

**Fig. 6:**
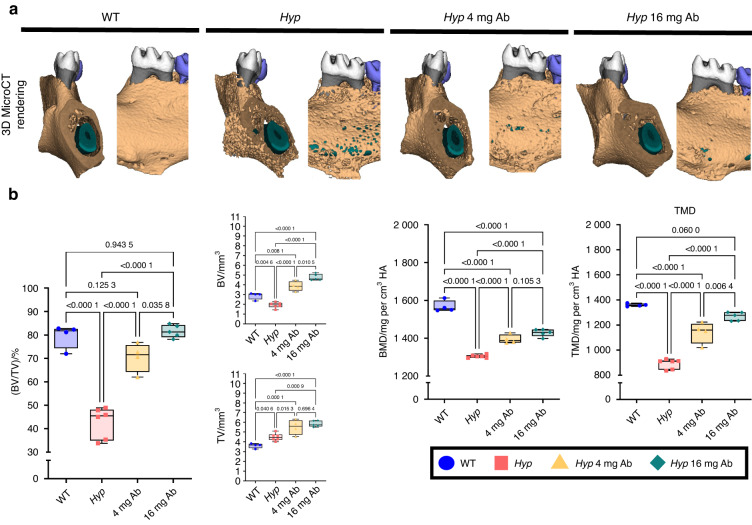
Impact of FGF23-mAb treatment on alveolar bone mineralization in *Hyp* mice. **a** Three-dimensional micro-computed tomography (mCT) present mandibular alveolar bone in the first molar region. **b** Treatment with both doses of FGF23-mAb improved alveolar bone volume fraction (BV/TV), bone mineral density (BMD) and total mineral density (TMD) in *Hyp* mice

**Fig. 7:**
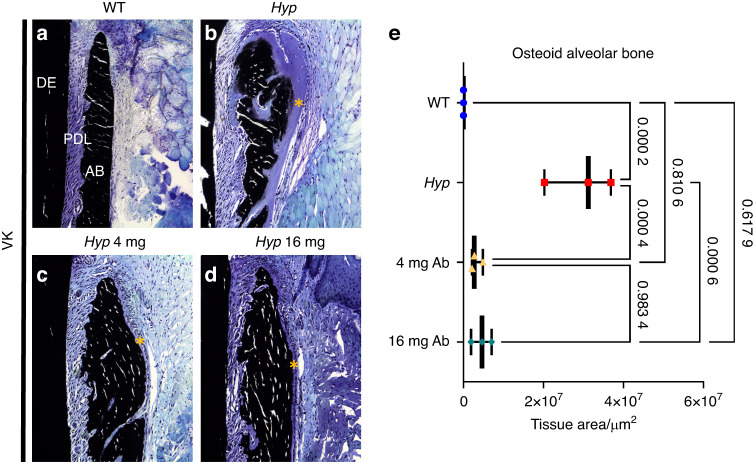
Histological analyses of the impact of FGF23-mAb therapy on bone in 3-month-old *Hyp* mice. **a**–**d** Von Kossa staining showed poor mineralization (in dark) of the collagen matrix revealing an increased amount of osteoid (yellow*) in *Hyp* mice alveolar bone. **e** Histomorphometry analysis demonstrated considerable osteoid reduction of alveolar bone after treatment with 4 mg or 16 mg·kg^−1^ of FGF23-mAb

The examination of the alveolar bone by micro-CT showed that *Hyp* animals treated with both doses of FGF23-mAb presented significantly increased bone volume fraction (BV/TV), bone mineral density (BMD), and tissue mineral density (TMD) (Fig. 6a, b) when compared to vehicle-treated *Hyp* mice. BV/TV of FGF23-mAb treated *Hyp* mice was similar to that of WT mice whatever the dose of the antibody (*P* = 0.125 3, *P* = 0.943 5, respectively). Furthermore, the analysis of the alveolar crest region demonstrated massive osteoid reduction after treatment with 4 mg·kg^−1^ or 16 mg·kg^−1^ of FGF23-mAb and therefore significant rescue of the alveolar crest mineralization (Fig. 7a–e).

### Treatment with FGF23-mAb corrects osteocyte number and reduces alveolar bone defects in *Hyp* Mice

**Fig. 8:**
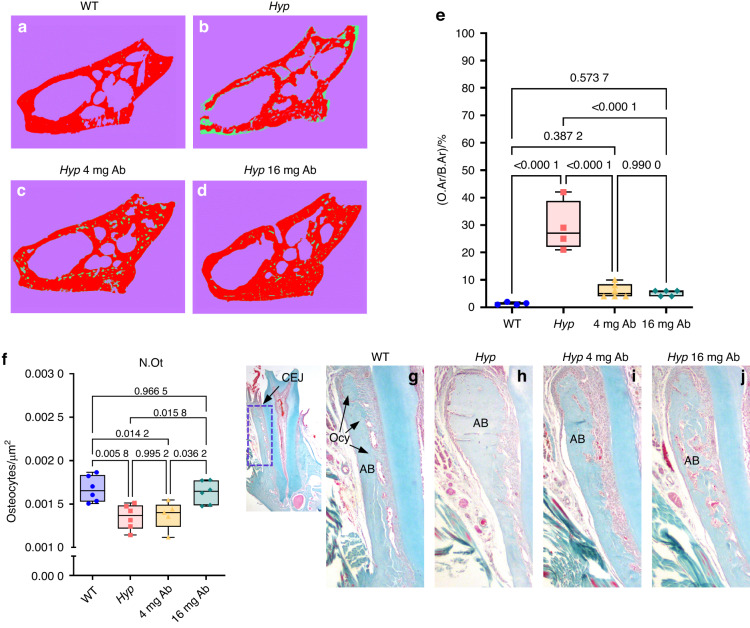
Histomorphometrical analysis of alveolar bone in FGF23-mAb treated *Hyp* mice. **a**–**d** Undecalcified sections automate segmented to measure osteoid in mice mandibles. **e** Histomorphometry analysis demonstrated a significant reduction of osteoid area/bone area (O.Ar/B.Ar) in alveolar bone after treatment with 4 mg or 16 mg·kg^−1^ of FGF23-mAb. **f** Alterations in osteocyte number (N.Ot) are observed in *Hyp* mice compared to WT mice, with full recovery by 16 mg·kg^−1^ FGF23-mAb treatment. **g**–**j** Masson’s Trichrome (MT) staining demonstrated distribution of osteocytes (Ocy) in alveolar bone and the region of interest (ROI). CEJ Cementum enamel junction

Examination by Von Kossa staining of the entire jawbone on frontal sections made at the level of the first molar furcation showed a 20% increase in the amount of osteoid (green color) versus fully mineralized bone (in red) in *Hyp* mice when compared to WT (Fig. 8a, b). In *Hyp* mice treated with both FGF23-mAb doses, we observed a dramatic decrease in osteoid accumulation, as well as improved bone mineralization similar to that of WT mice (Fig. 8a–e), consistent with our micro-CT measurements (Fig. 6a, b). We next calculated the number of osteocytes in the alveolar crest in the different groups of animals and were able to show that vehicle-treated *Hyp* mice and 4 mg·kg^−1^ FGF23-mAb treated *Hyp* mice displayed a lower osteocyte number (N.Ot) in comparison to WT animals. In contrast, the number of osteocytes was fully recovered in *Hyp* mice treated with 16 mg·kg^−1^ of FGF23-mAb (Fig. 8f–j).

### Expression of alveolar bone and acellular cementum markers is restored by FGF23-mAb treatment

**Fig. 9:**
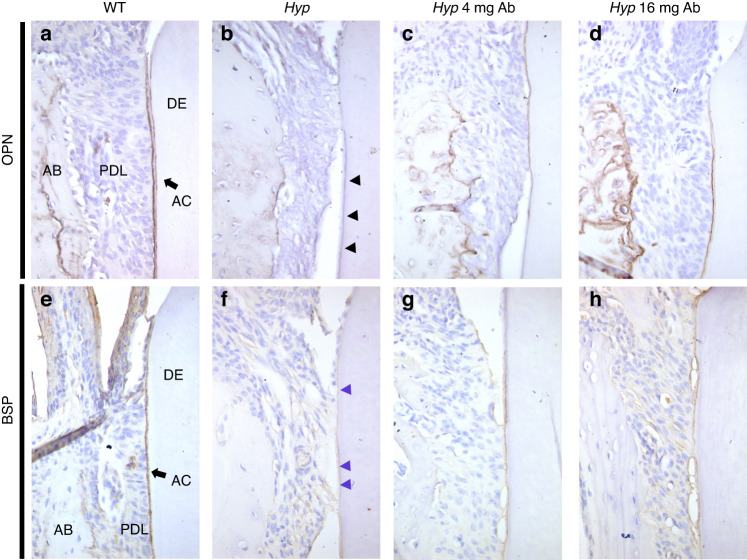
Expression of bone and cementum markers in FGF23-mAb treated *Hyp* mice. **a**–**d** Osteopontin (OPN) is a marker of acellular cementum (AC) and alveolar bone (AB), and all groups presented OPN staining on root surfaces, even though *Hyp* mice have a defective pattern (black arrows). Both treatments showed improved localization of OPN in acellular cementum. OPN is not similarly distributed in *Hyp* versus WT alveolar bone, and OPN is present in *Hyp* mice alveolar bone with both doses of treatment, showing a localization at the bone surface and around osteocytes. **e–h** Bone sialoprotein (BSP) is a marker of AC and AB. AC defects in *Hyp* mice are shown as irregular BSP staining (purple arrows), however, both 4 mg or 16 mg·kg^−1^ of FGF23-mAb improve BSP localization on root dentin surfaces

We next evaluated osteopontin (OPN) and bone sialoprotein (BSP) expression in the *Hyp* anchorage apparatus, as these proteins are known as classical markers for cementum and bone^26,27^ (Fig. 9). As expected, strong and continuous OPN (Fig. 9a, b) and BSP (Fig. 9e, f) immunolabelings were observed along the molar root in the acellular cementum of WT mice whereas these labelings were fainter and disrupted in *Hyp* mice; this is consistent with the abnormal acellular cementum previously reported in humans with XLH^6^ and in *Hyp* mice.^9^ In animals treated with both FGF23-mAb doses the localization and the intensity of OPN and BSP immunolabeling was improved (Fig. 9c, d, g, h, respectively), with a pattern similar to that of WT mice for the higher dose of FGF23-mAb treatment.

In the alveolar bone, OPN immunolabeling was seen at the bone surface and in the vicinity of osteocyte lacunae in WT animals. In the vehicle-treated *Hyp* mice, this staining was prominently localized in the bone extracellular matrix. The FGF23-mAb treated *Hyp* mice showed persistent accumulation of OPN in the alveolar bone crest (Supplementary Fig. 3). Both FGF23-mAb treatment doses restored the localization of OPN at the bone surface and around osteocytes in *Hyp* mice (Fig. 9a–d). BSP staining in the alveolar bone of *Hyp* mice showed a pattern of erratic distribution in the extra cellular matrix compared to WT mice as reported by others^28^; this irregular distribution was partially corrected in *Hyp* mice treated with both FGF23-mAb doses (Fig. 9e–h).

## Discussion

Our study demonstrates that repeated FGF23-mAb injections in young *Hyp* mice, the murine model that replicates the skeletal features associated with XLH, improves dentin and alveolar bone mineralization.^9,28–30^ FGF23-mAb markedly improved dentin mineralization of the first molar, as evidenced by a decrease in the predentin width and a correction of the volume of the pulp chambers, two hallmarks features of XLH.^4^ Furthermore, FGF23-mAb treatment attenuated the periodontal tissues defects reported in the XLH acellular cementum,^28,31^ improved the PDL attachment and the cellular cementum mineralization with a decrease in non-mineralized cementum (cementoid). In addition, FGF23-mAb therapy restored the alveolar bone microarchitecture parameters, decreased the amount of osteoid tissue, and promoted bone mineralization. Noteworthy, the improvement of these features was greater in *Hyp* mice treated with the highest dose of FGF23-mAb. In other words, *Hyp* mice treated with 16 mg·kg^−1^ of FGF23-mAb exhibited a complete rescue of the dentoalveolar tissue defects related to XLH. Hence, these findings support the efficacy of the FGF23-mAb therapy in *Hyp* mice on the dentoalveolar tissues and show that these outcomes are dose-dependent, as previously demonstrated for the rest of the skeleton.^13^ This suggests that FGF23-mAb treatment may improve dentin features not only in juvenile^16,21^ but also in young adults, as supported by both our murine and clinical data. Our study also showed that the first molar of the *Hyp* mice is a robust model to study tooth mineralization rather than the continuously growing incisor, as it exhibits most of the dental features previously reported in patients with XLH.^6,32^

A recent study analyzed the impact of XLH treatments on dentoalveolar tissues in *Hyp* mice, including a therapy targeting FGF23.^23^ In contrast with the positive outcomes reported here, this therapy showed a limited impact on alveolar bone and dental features, failing to demonstrate additional benefits compared to 1,25-(OH)_2_-vitamin D3 treatment. These discrepancies might be due to important differences between the two studies, such as dose and origin of the antibody (35 mcg/g; Amgen, Thousand Oaks, CA in 27), and age at onset and duration of the treatment (from 2 days to 35 days after birth, 3 time a week with the FGF23-blocking antibody).^23^ Notably, the antibody administrated in our study is the one used to develop burosumab and our clinical data further support the positive impact on dental and bone tissues (Fig. 1). There are currently two treatment options for XLH available in the clinic: (1) conventional treatment consisting of supplementation of phosphorus and active vitamin D analogs and (2) burosumab, which directly targets the elevation of FGF23. The therapeutic strategies and targets are different, but both seem to provide different degrees of improvement regarding dental and alveolar bone tissues.^23^ In the future, it is recommended that the evaluation of different treatments be continued in the same animal studies to provide more reliable comparisons.

Our results demonstrated increased dentin volume as an outcome of FGF23-mAb treatment while dentin density was not improved in treated *Hyp* mice. This may be due to the fact that part of the molar dentin formed before the onset of treatment and that its total density is not fully rescued by the treatment as, contrary to bone, dentin is not remodeled.^33^ Together with qualitative observations, quantitative analysis of dentin mineralization might provide further clues on whether or how dentin is involved in the development of abscesses. Given the fact that FGF23-mAb promoted consistent improvements in *Hyp* mouse dentin formation, burosumab administration might be associated with decreased dental abscesses, as recently indicated in a monocentric clinical trial.^21^ Abnormal dentin mineralization together with enlarged pulp chambers is considered as the main culprit for the high incidence of dental abscesses in XLH patients.^33–36^ The randomized phase 3 clinical trials conducted in children^37^ or in adults^38^ with XLH did not report the benefit of burosumab on the dental abscess. However, it is important to note that patients were treated with burosumab for a short period of time during these clinical trials (64 weeks and 24 weeks, respectively). Therefore, the dental infections reported in these trials occurred on teeth that mineralized long before the onset of burosumab treatment. Interestingly, a post hoc analysis of the pediatric clinical trial^37^ reported that the group of young children treated with burosumab were less susceptible to dental abscesses than the age-matched patients who received the conventional therapy. This observation was not found in older children treated later with the biotherapy, suggesting a window of opportunity during tooth formation to positively limit the risk of abscess formation.^17^ Our recent retrospective study dedicated to the dental follow-up of children with XLH confirmed that burosumab improved tooth mineralization in children as it was associated with a reduction in the number of dental abscesses in comparison to the conventional treatment.^21^

Periodontium defects may lead to important loss of function including tooth protection, microbiome homeostasis, and tooth anchorage. Our histological analysis revealed altered tooth attachment in the *Hyp* mice, consistent with previous observations in mice^9^ and humans.^6^ This deficiency of the periodontium may explain the higher susceptibility to periodontal disease of adult patients with XLH.^6,39^ Here, both FGF23-mAb doses partially rescued the *Hyp* PDL attachment, with improved acellular cementum continuity associated with the restoration of BSP and OPN markers expression, leading to PDL fibers insertion to connect the alveolar bone and the tooth root. Therefore, the ability of FGF23-mAb treatment to improve acellular cementum mineralization may be driven by the adjusted expression of non-collagenous matrix proteins such as SIBLINGs, particularly BSP and OPN well-acknowledged markers of acellular cementum mineralization.^26^ Our results demonstrated significant improvement on alveolar bone microarchitecture and mineralization, however OPN accumulation in *hyp* mice alveolar bone persists despite the anti-FGF23 treatment. These findings suggest that targeting FGF23 elevation does not fully correct extracellular matrix composition. Future studies are needed both in *Hyp* mice and, at a longer scale, in humans, to explore the impact of FGF23 inhibition on the ECM proteins present in the treated dental tissues compared to untreated animals^40^ and patients.^41^ Overall, the benefits in the periodontium-supporting structures observed in treated mice suggest that burosumab may decrease the susceptibility of adult patients to periodontitis. This needs to be evaluated in the longer term. In addition, improved bone quality may be critical to the clinical outcomes of orthodontic and implant treatments in XLH patients.

*PHEX* inactivation leads to pathological elevation of FGF23 (fibroblast growth factor 23), a hormone synthesized by osteocytes and osteoblasts. FGF23 is stimulated by the active form of vitamin D (calcitriol) and phosphate and inhibited by Dentin Matrix acidic Phosphoprotein 1 (DMP1). DMP1 binds to PHEX via an ASARM (Acidic Serine- and Aspartate-Rich MEPE-associated) motif resulting in the downregulation of FGF23.^42^ FGF23 can be modified in two ways: (1) Phosphorylated by FAM20c, FGF23 is cleaved by furin leading to the release of N-FGF23 and C-FGF23 fragments; (2) Glycosylated by GALNT3, intact FGF23 circulates and binds to the FGFR1 (receptor) and α-klotho (co-receptor) in renal cells. This leads to the withdrawal of the sodium/phosphate transporters NaPi2a/c from the renal epithelial cell membrane, resulting in urinary phosphate wasting. FGF23 also plays a role in vitamin D metabolism, via inhibition of CYP27B1, a 1α-hydroxylase activating synthesis of calcitriol and upregulating 24-hydroxylase that breaks down the active form of 1,25-dihydroxyvitamin D_3_.^43^

FGF23 blocking acts neutralizing the full length of FGF23. From the dentoalveolar point of view, we believe improvements related to anti-FGF23 are related to increased local levels of phosphate and active vitamin D, which has a huge impact on cementum mineralization.^35,44^ In dentin, SIBLING proteins and pASARM may play a more important role.^45^ Hower, the development of bone and dentin is close and start early in timeline, while deposition of cementum by cementoblasts occurs later in tooth development. Our results demonstrate complete rescue in architecture and cementum markers localization. This may be explained by the precocious time of initiation of the treatment regarding cementum formation compared to dentin. Despite the differences between tooth (enamel, dentin, cementum) and bone development, mineralization of those tissues involves similar molecular processes and is often affected by similar molecular mechanisms. The canonical Wnt signaling pathway is associated to the regulation of bone homeostasis^46^ and odontogenic differentiation impacting dentin formation and mineralization in XLH patients.^47^

In conclusion, this study showed that alveolar bone and dental tissues are highly responsive to FGF23-mAb treatment, in a dose-dependent manner. Substantial improvement with both 4 mg·kg^−1^ and 16 mg·kg^−1^ doses of FGF23-mAb in *Hyp* animals supports further studies of the underlying disease-related mechanisms together with functional approaches to challenge the treated tissues. Overall, the positive impact of FGF23-mAb on dentoalveolar features in *Hyp* mice at a short time scale heralds the efficacy of burosumab on oral tissues in humans. It is now mandatory to collect clinical data including dental and periodontal evaluation from XLH patients to confirm the benefit of this therapy on oral manifestations.

## Materials and methods

### X-Ray and Cone-beam computed tomography (CBCT)

X-ray and CBCT scans (Planmeca ProMax® 3D Max, Helsinki, Finland) were collected from two XLH patients imaged before and after burosumab treatment according to French law (loi Jardé) as detailed in the Supplementary Materials. Morphometric tooth measurements and bone analysis are detailed in the Supplementary Materials.

### Mice

All animal studies were performed in accordance with Standards for Proper Conduct of Animal Experiments at Kyowa Kirin Co., Ltd., Japan and followed the Animal Research: Reporting of In Vivo Experiments (ARRIVE) guidelines. Thirty-one mice on a C57BL/6 J background were randomly (Random number calculators; GraphPad) divided into four groups *n* = 3–7 mice per group. Male *Hyp* mice from four weeks to 3 months were subcutaneously injected weekly with an FGF23-neutralizing antibody (FGF23-mAb, 4 mg·kg^−1^ or 16 mg·kg^−1^; Kyowa Kirin). WT and *Hyp* control littermates received PBS (-) injections weekly as a vehicle. Doses were based on previous reports that demonstrated the efficacy of anti-FGF23-mAb in mice.^13^ Serum biochemistries were analyzed before harvesting, Phospha C-Test Wako (FUJIFILM Wako Pure Chemical Corporation) kit was used to measure mice serum phosphate. Additional details are in the supplementary material (Supplementary Fig. 4).

### Micro–Computed Tomography

Hemimandibles were scanned using an X-ray micro-CT device (Quantum FX Caliper, Life Sciences, Perkin Elmer, Waltham, MA, United States) at 90 kVp, 160 µA, 180 s integration time, and 10-µm voxel dimension. Analyses were performed as previously described^23,48^ for *n* = 4–6 samples per experimental group.

### Histology

Hemimandibles were demineralized (*n* = 4–6 mice per group) in 4% EDTA solution, then embedded in paraffin for 6-µm serial sectioning. Coronal sections were stained by Masson’s trichrome, toluidine blue, or picrosirius red.^9^ Undecalcified samples (*n* = 3 mice per group) were embedded in methyl methacrylate (Merck, Rahway, NJ) and 4-μm thickness sections were obtained. Non-decalcified Von Kossa staining was performed for bone/osteoid and cementum/cementoid measurements as detailed in the Supplementary Materials. Histomorphometry for bone, cementum and predentin and immunohistochemistry (IHC) are described in the Supplementary Materials. All the measurements were performed by two blind evaluators.

### Statistical analysis

Data were checked for normality and equal variance and analyzed using one-way analysis of variance (ANOVA) with post hoc Tukey test and clinical data was analyzed using paired *t* test (Prism version 9.0; GraphPad Software), where *P* < 0.05 was considered statistically significant.

### Supplementary information

Supplemental Material

Supplemental Fig. 1

Supplemental Fig. 2

Supplemental Fig. 3

Supplemental Fig. 4

## Data Availability

All data associated with this study are presented in the paper.
