# Supplementary material for: Dental impact of anti-fibroblast growth factor 23 therapy in X-linked hypophosphatemia
Source: Int J Oral Sci. 2023 Dec 6;15:53. doi: 10.1038/s41368-023-00259-8 (PMC10697996; doi:10.1038/s41368-023-00259-8)
Supplement: Supplementary file 1 — Supplemental Material [file 41368_2023_259_MOESM1_ESM.docx]

**SUPPLEMENTARY INFORMATION**

**Dental impact of anti-FGF23 therapy in X-linked hypophosphatemia**

**Running title:** Dental impact of FGF23 antibody in *Hyp* mice

Elis J. Lira dos Santos^1^, Kenta Nakajima^2^, Julien Po^1^, Ayako Hanai^2^, Volha Zhukouskaya^1^, Martin Biosse Duplan ^3,6^, Agnès Linglart^4^, Takashi Shimada^5^, Catherine Chaussain^1,4^, Claire Bardet^1^

^1^Université Paris Cité, Institut des maladies musculo-squelettiques, Laboratory Orofacial Pathologies, Imaging and Biotherapies URP2496 and FHU-DDS-Net, Dental School, and Plateforme d’Imagerie du Vivant (PIV), Montrouge, France

^2^R&D Division, Kyowa Kirin, Co., Ltd, 3-6-6 Asahi-machi, Machida-shi, Tokyo 194-8533, Japan

^3^ Université Paris Cité, Institut Imagine, INSERM UMR 1163, Paris, France

^4^Paris-Saclay University, AP-HP, INSERM U1185, DMU SEA, Endocrinology and Diabetes for Children, Reference Center for Rare Diseases of the Calcium and Phosphate Metabolism, OSCAR filière, EndoRare, and BOND ERNs, Bicêtre Paris Saclay Hospital, Le Kremlin-Bicêtre, France

^5^Medical Affairs Department, Kyowa Kirin, Co., Ltd, 1-9-2 Otemachi, Chiyoda-ku, Tokyo 100-0004, Japan

^6^AP-HP, Reference Center for Rare Disorders of the Calcium and Phosphate Metabolism, Dental Medicine Department, Bretonneau Hospital, GHN-Université Paris Cité, Paris, France

**MATERIALS AND METHODS**

**Regulatory approval for the use of human medical data**

This study was designed, conducted, recorded, and reported in accordance with the principles established by the World Medical Association Declaration of Helsinki Ethical Principles for Medical Research Involving Human Subjects.

The two participants were verbally informed about the potential use of their anonymized medical data for research purposes and the participants' non-objection was collected. The participants had the right to withdraw from the study at any time by completing the form available from a link at: <http://recherche.aphp.fr/eds/droit-opposition>. According to French law (loi Jardé), anonymous monocentric retrospective studies do not require institutional review board approval.

**X-Ray and Cone-beam computed tomography (CBCT)**

X-ray and CBCT scans (Planmeca ProMax® 3D Max, Helsinki, Finland) were collected from two XLH patients imaged before and after burosumab treatment as part of routine care and indicated exclusively by the patients clinical conditions. Morphometric tooth measurements on x-rays were performed using imageJ version 1.46r (National Institutes of Health, Bethesda, MD). For the CBCT analysis, the areas of the mandibular premolars were cropped in each 2D section. Calculation of the 3D structural parameters followed the guidelines published by the American Society for Bone and Mineral Research ^1^. Reconstructed DICOM images of bone were analyzed using AnalyzePro version 1.0 (AnalyzeDirect, Overland Park, KS).

**Micro-computed Tomography (micro-CT)**

Hemi-mandibles were scanned in X-ray micro-CT device (Quantum FX Caliper, Life Sciences, Perkin Elmer, Waltham, MA, United States) at 90 kVp, 160 µA, 180 s integration time, and 10-µm voxel dimension. Reconstructed images were analyzed using Analyze 14.0 (AnalyzeDirect, Overland Park, KS) as previously described ^2^.

*Calibration*

For proper conversion of grayscale numbers to mineral density units (mg/cm^3^HA) a phantom was scanned with the image data using the same acquisition protocol. We used a phantom with five Known mineral content (0, 50, 200, 800 and 1200 mg/cm3). We plotted the grayscale value versus mineral density of the phantom and created a linear regression of mean grayscale value versus phantom mineral density. DICOM files were calibrated according to recorded slope and intercept values.

*Orientation*

Hemi-mandibles (n=4-6 per experimental group) were oriented using first molar anatomical landmarks. In the sagittal plane, samples were oriented respecting the mesial and distal portions of the cementoenamel junction (CEJ) and complete open root canal in the middle of the second third of mesial and distal roots. In the coronal view, we oriented the samples according to the long axis of the root canal. Finally, in the axial plane orientation was defined by the center of the mesial and distal root canals.

*Region of Interest (ROI)*

The region of interest (ROI) for alveolar bone analysis was defined after orientation to ensure proper comparison of all samples. First mandibular molar mesial and distal edges of the crown were used as anatomical landmarks on the sagittal plane. To include increased amount of bone tissue we expanded the analyses 400 μm mesial to the mesial edge and 400 μm distal to the distal edge, therefore including all bone on buccal and lingual sides. For enamel, dentin/cementum and pulp chamber we included data exclusively related do the mandibular first molar.

*Segmentation*

For segmentation of the dentoalveolar tissues we applied threshold values. First, enamel was segmented at 1600 mg/cm^3^HA. Dentin and cementum are challenging to be accurately segmented in *Hyp* mice ^3,4^ because of the massive mineralization defects, therefore those two tissues were analyzed in combination as reported previously ^5,6^. Dentin/cementum and bone were segmented at 450 mg/cm^3^HA.

**Histology**

*Undecalcified samples*

Hemimandibles were dissected (*n*=3) and fixed at 4°C in 70% ethanol (2 days) and dehydrated in graded ethanol series (15 days), the mandibles were embedded in methyl-methacrylate (Rahway, NJ, United States), and polymerized at 20°C for 48 h. After polymerization, high density tissue such as enamel was removed from the tooth crown to ensure proper cut of other dentoalveolar tissues. Prepared samples were sectioned in a Polycut E microtome (Leica, Wetzlar, Germany). Serial sections 4 μm thickness were obtained in the coronal plane of the first (eg. molar long axis) and stained with von Kossa (5% silver nitrate solution, Sigma-Aldrich, St Louis, MO, United States) and counterstained with toluidine blue (pH 3.8).

*Decalcified samples*

Hemimandibles (*n*= 4–6 mice/group) were fixed in 10 % neutral buffered formalin and demineralized in 4% EDTA solution at room temperature with agitation for 4 weeks, then embedded in paraffin for 6-µm serial sectioning. Deparaffinized coronal sections of mandibles in the first molar region were stained by Masson’s trichrome, toluidine blue, or picrosirius red ^7^.

**Histomorphometry**

Histomorphometry was performed on representative sections from n=4-8 mice/experimental group. Thicknesses of PDL and predentin was measured at 90, 100, and 110 μm apical to the cementum-enamel junction (CEJ) on the buccal side of first molar in central coronal sections of the mesial root, and values were averaged for each sample. PDL attachment defect was measured in first molar in central coronal sections of the mesial root, from the CEJ to end of the second third of the root (beginning of cellular cementum). The length of defective areas was average for each animal and the percentage of attachment defect was obtained.

Cellular cementum area was measured in central coronal sections of the mesial root, combining buccal and lingual cellular cementum area measurements to a total area. Measurements were made with ImageJ version 1.8.06 (National Institutes of Health, Bethesda, MD).

To analyze bone mineralization in the mandible, Von Kossa-stained sections were used to distinguish the mineralized bone (black) from non-mineralized-bone matrix (purple). Trainable Weka Segmentation (TWS), an open-source plugin on Image J, was used to determine the mineralized area as previously described ^8^. Central furcation coronal sections of the mandibular first molar was used for this quantification.

For each image three classes were created on the “settings” option on the TWS window: “Mineralized Bone”, “non-mineralized bone” and “background”, at least 10 points for each class were marked with the freehand tool of ImageJ and associated to each class by “add to class” option. The classifier software was calibrated until the segmentation was accurate. And the final image has been created by the TWS where each class was color coded. We refined the results by removing the source of background noise and the incisor tooth. We used Color Inspector 3D, another plugin of ImageJ to quantify the area of each class. Additionally, we performed osteoid quantification in the buccal aspect of the alveolar bone in central coronal sections of the mesial root from the CEJ to 110 μm apical.

For quantification of osteocyte, we used alveolar bone (n=5-6 mice). Alveolar bone regions included buccal alveolar bone from the CEJ to the apical portion of the first mandibular molar mesial root. Osteocyte number was defined by the number of osteocytes normalized to the total bone area in each sample. Total bone area (whole area) was traced using the freehand selection function in ImageJ.

**Immunohistochemistry**

Immunohistochemistry (IHC) was performed using an avidin-biotinylated peroxidase-based kit with a 3-amino-9-ethylcarbazole substrate (Vector Labs, Burlingame, CA) to produce a red-brown product (n = 3 animals/genotype). Primary antibodies included rabbit polyclonal anti-bone sialoprotein 1:100 (LF-84 BSP; Kerafast, Boston, MA) and goat polyclonal anti-human osteopontin 1:100 (AF1433; OPN, R&D Systems, Minneapolis, MN).

**REFERENCES**

1. Bouxsein, M. L. *et al.* Guidelines for assessment of bone microstructure in rodents using micro-computed tomography. *Journal of Bone and Mineral Research* **25**, 1468–1486 (2010).

2. Chavez, M. B. *et al.* Guidelines for Micro-Computed Tomography Analysis of Rodent Dentoalveolar Tissues. *JBMR Plus* **5**, e10474 (2021).

3. Ao, M. *et al.* Overlapping functions of bone sialoprotein and pyrophosphate regulators in directing cementogenesis. *Bone* **105**, 134–147 (2017).

4. Thumbigere-Math, V. *et al.* Hypercementosis Associated with ENPP1 Mutations and GACI. *J Dent Res* **97**, 432–441 (2018).

5. Zhang, H. *et al.* Dentoalveolar Defects in the Hyp Mouse Model of X-linked Hypophosphatemia. *J Dent Res* **99**, 419–428 (2020).

6. Lira dos Santos, E. J. *et al.* Effects of Active Vitamin D or FGF23 Antibody on Hyp Mice Dentoalveolar Tissues. *J Dent Res* **100**, 1482–1491 (2021).

7. Coyac, B. R. *et al.* Tissue-specific mineralization defects in the periodontium of the Hyp mouse model of X-linked hypophosphatemia. *Bone* **103**, 334–346 (2017).

8. Malhan, D. *et al.* An Optimized Approach to Perform Bone Histomorphometry. *Front Endocrinol (Lausanne)* **9**, 1–11 (2018).

**FIGURE CAPTIONS**

**Supplemental Figure 1. Enamel is not affected in the murine model of XLH.** **(A-H)** Two and 3-dimensional micro-computed tomography (mCT) showing the enamel of the mandibular first molar (E). **(I, J)** Enamel volume and density were measured by mCT and no significant difference was detected in any of the groups.

**Supplemental Figure 2. Impact of FGF23-mAb on incisor teeth in the *Hyp* mouse**. **(A-H)** Rendering and 2-dimensional micro-computed tomography (mCT) showing mandibular incisor dentin (De), pulp chamber (Pu) and enamel (E). **(I)** Enamel and dentin parameters of volume and density were not different. **(J)** Toluidine blue staining (under first molar region) reveals wide predentin (PD) (yellow asterisk) and interglobular DE patterns in *Hyp* mice (red arrow). Dentin and predentin are fully normalized by either FGF23-mAb treatments.

**Supplemental Figure 3. Alveolar bone marker in the *Hyp* mouse**. **(A, B)** Osteopontin (OPN) intensity and distribution in the WT appears more regular compared to *Hyp* mice. **(C, D)** Treated alveolar bone showed OPN accumulation predominantly in the edges of osteocytes lacunae. Osteopontin distribution is not fully normalized by either FGF23-mAb treatments.

**Supplemental Figure 4. Biochemistry results of *Hyp* mice following FGF23-mAb treatment.**  Serum inorganic phosphate biochemical results in treatment groups (n=10 mice per experimental group).
