# Supplementary figures and images for: Dental impact of anti-fibroblast growth factor 23 therapy in X-linked hypophosphatemia
Source: Int J Oral Sci. 2023 Dec 6;15:53. doi: 10.1038/s41368-023-00259-8 (PMC10697996; doi:10.1038/s41368-023-00259-8)

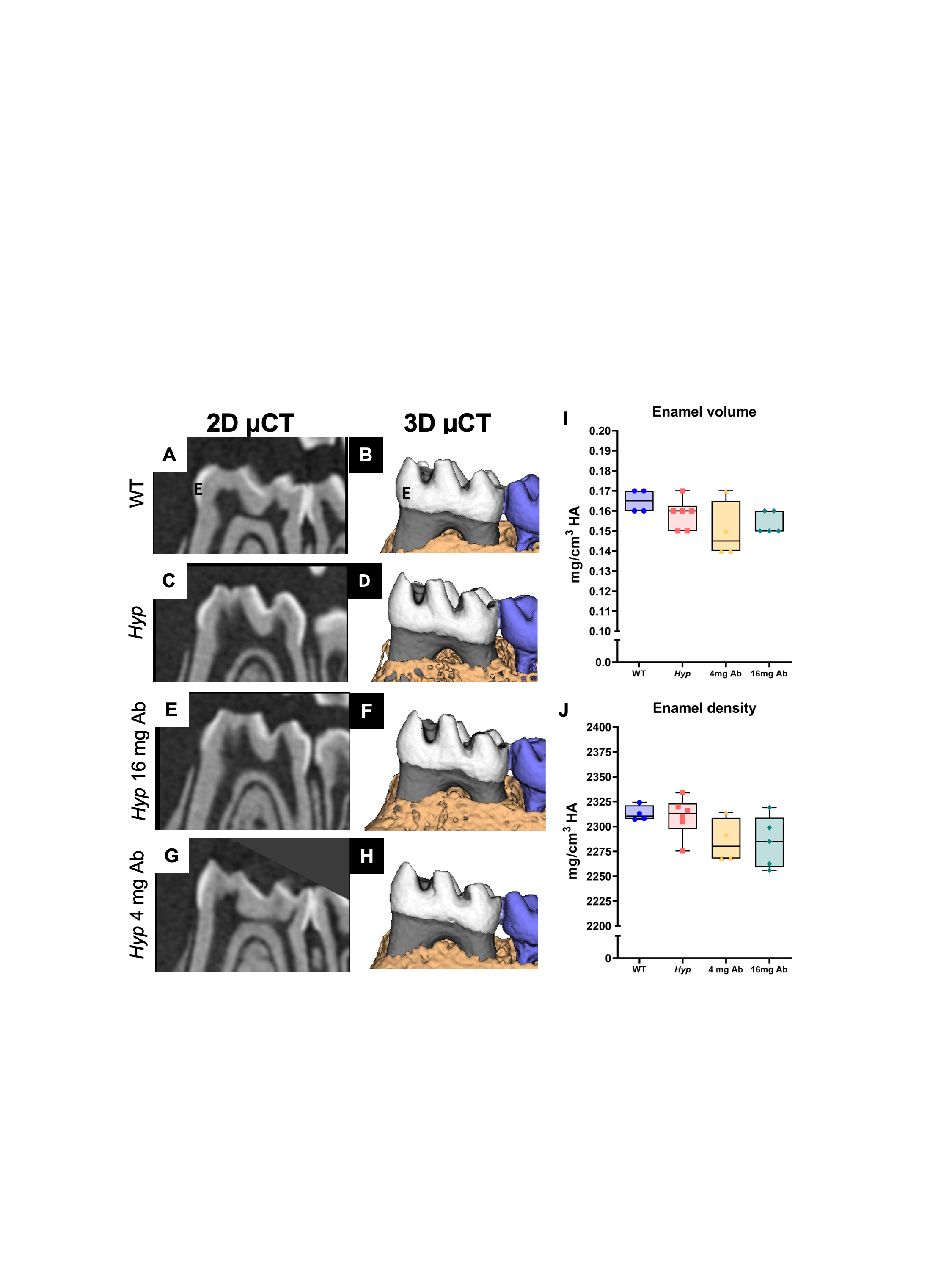

Supplement: Supplementary file 2 — Supplemental Fig. 1 [file 41368_2023_259_MOESM2_ESM.tif]

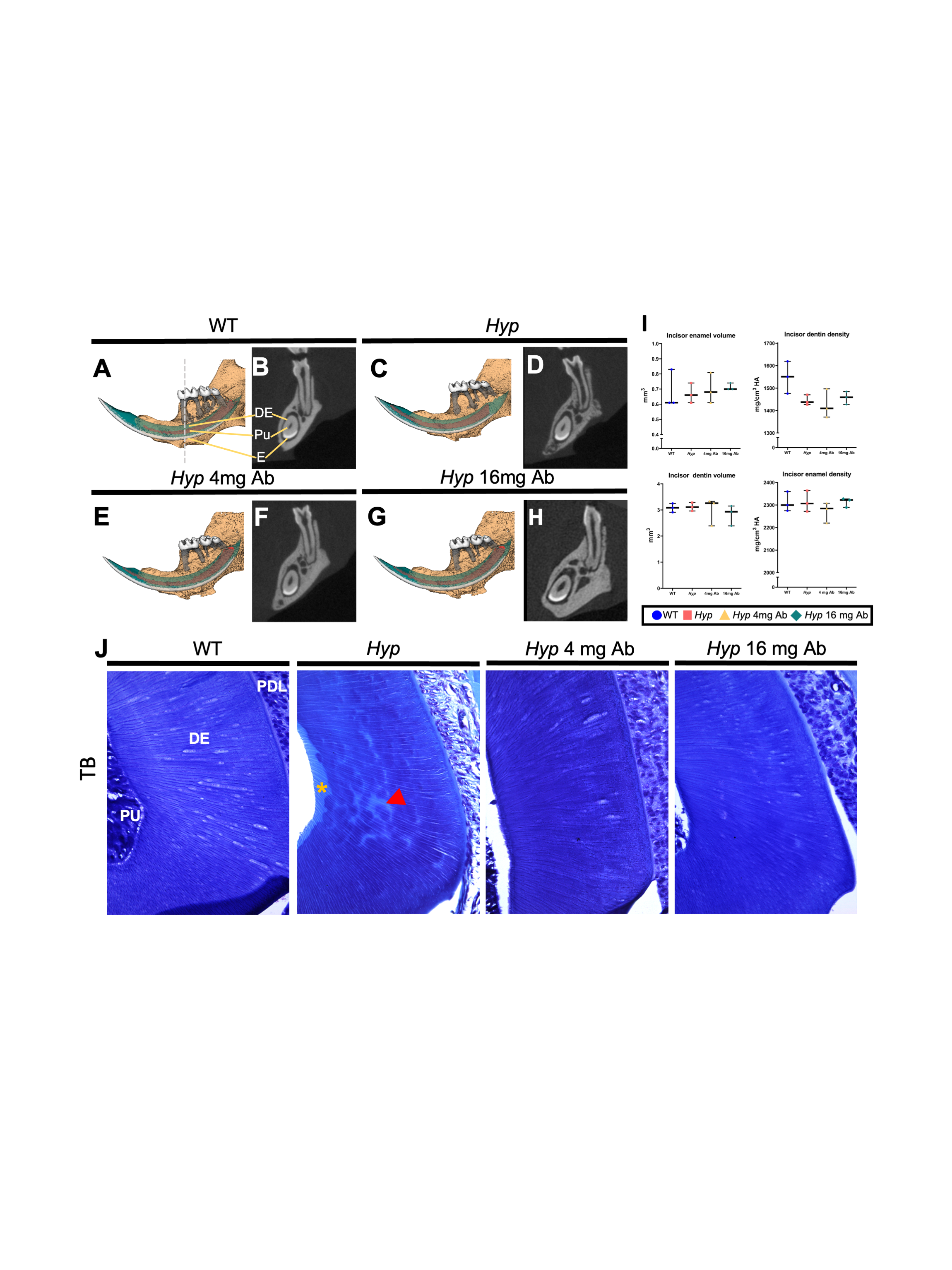

Supplement: Supplementary file 3 — Supplemental Fig. 2 [file 41368_2023_259_MOESM3_ESM.tif]

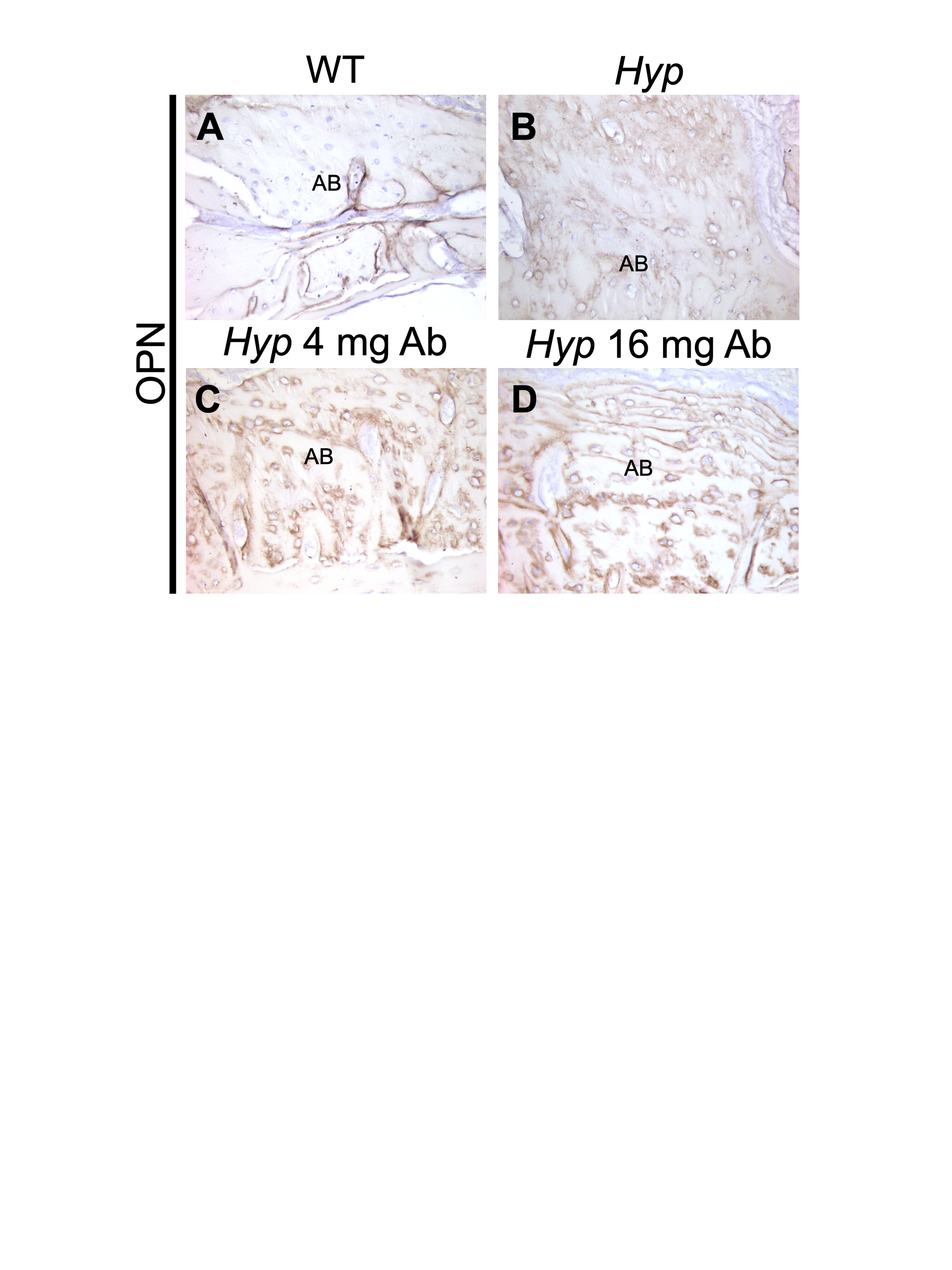

Supplement: Supplementary file 4 — Supplemental Fig. 3 [file 41368_2023_259_MOESM4_ESM.tif]

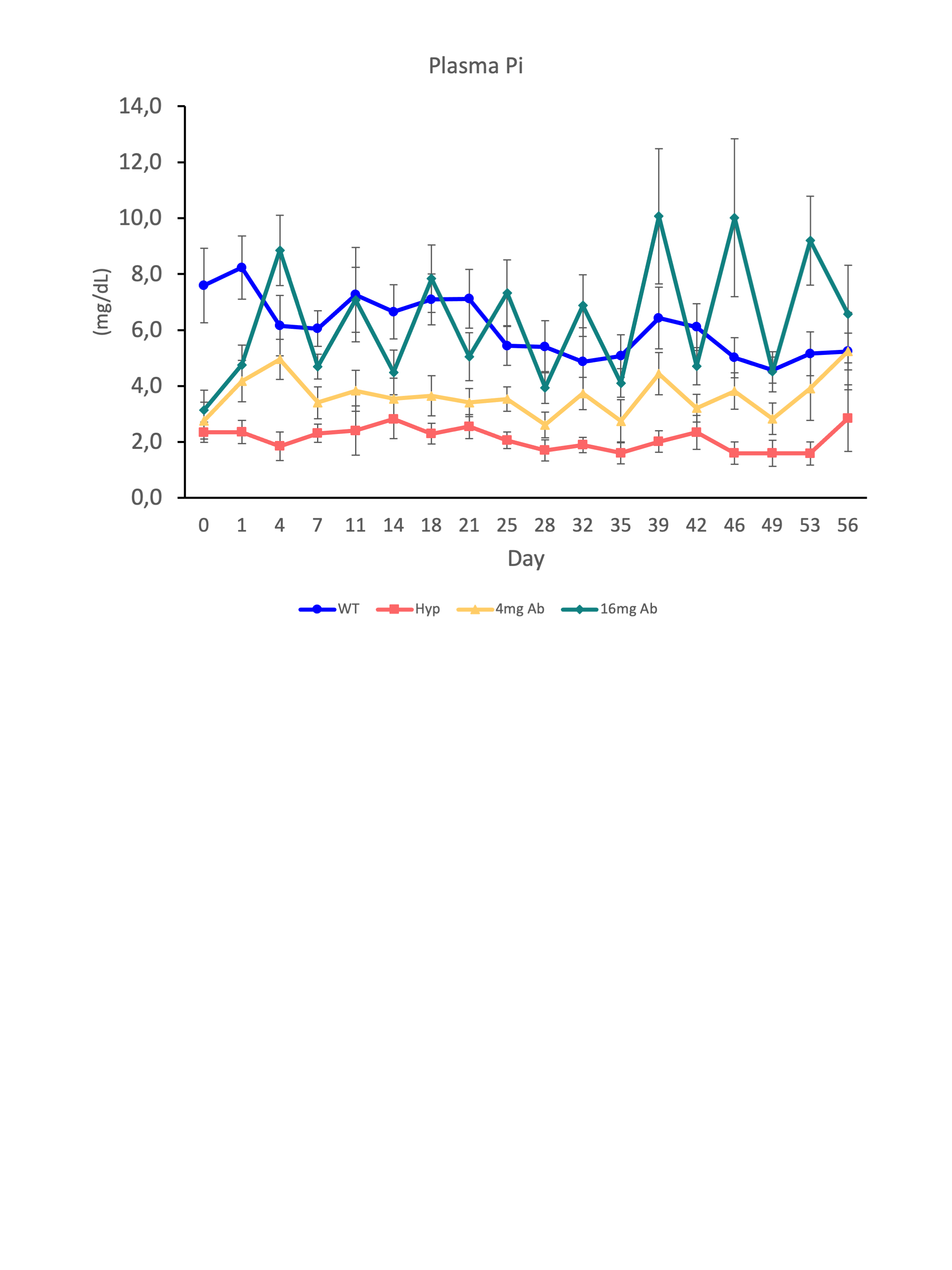

Supplement: Supplementary file 5 — Supplemental Fig. 4 [file 41368_2023_259_MOESM5_ESM.tif]
